# Effect of a Stannous Fluoride Dentifrice on Biofilm Composition, Gene Expression and Biomechanical Properties

**DOI:** 10.3390/microorganisms10091691

**Published:** 2022-08-23

**Authors:** Hardeep Kaur Gumber, Artemis S. Louyakis, Tulika Sarma, Kristina Ivana Fabijanic, Reeba Paul, Kristen Mellenbruch, Latonya Kilpatrick-Liverman

**Affiliations:** 1 Colgate-Palmolive Technology Center, Piscataway, NJ 08854, USA; 2 Hill’s Pet Nutrition, Inc., Topeka, KS 66603, USA

**Keywords:** oral biofilm, stannous fluoride, dental plaque, oral health, oral microbiome, toothpaste

## Abstract

An in situ study was conducted to examine the mode of action of a 0.454% stannous fluoride (SnF_2_)-containing dentifrice in controlling the composition and properties of oral biofilm. Thirteen generally healthy individuals participated in the study. Each participant wore an intra-oral appliance over a 48-h period to measure differences in the resulting biofilm’s architecture, mechanical properties, and bacterial composition after using two different toothpaste products. In addition, metatranscriptomics analysis of supragingival plaque was conducted to identify the gene pathways influenced. The thickness and volume of the microcolonies formed when brushing with the SnF_2_ dentifrice were dramatically reduced compared to the control 0.76% sodium monofluorophosphate (MFP)-containing toothpaste. Similarly, the biophysical and nanomechanical properties measured by atomic force microscopy (AFM) demonstrated a significant reduction in biofilm adhesive properties. Metatranscriptomic analysis identified pathways associated with biofilm formation, cell adhesion, quorum sensing, and N-glycosylation that are significantly downregulated with SnF_2_. This study provides a clinically relevant snapshot of how the use of a stabilized, SnF_2_ toothpaste formulation can change the spatial organization, nanomechanical, and gene expression properties of bacterial communities.

## 1. Introduction

Currently, the Centers for Disease Control has reported that close to 50% of all US adults of 30 years or older has some form of gum disease, with the percentage of incidents increasing to 70% among adults 65 years or older [1]. The early stage of the disease is called gingivitis, where bacterial infection and host response leads to swollen and bleeding gums. If left untreated, the disease progresses to a more serious and irreversible form called periodontitis, where gum recession and bone detachment lead to tooth loss [2]. Periodontal disease has been associated with cardiovascular disease, diabetes mellitus, and adverse pregnancy outcomes, implicating oral health as an important factor in systemic health [3]. The primary factor contributing to gum disease and tooth decay is plaque accumulation on teeth [4]. Dental plaque or the oral biofilm is formed by the three-dimensional organization of microorganisms on the oral surfaces. The microorganisms are embedded in the exo-polysaccharide matrix secreted by its inhabitants [5]. The exo-polysaccharide matrix is comprised of sugars, proteins, lipids, and extracellular nucleotides. The matrix provides architecture, strength, and protection to the embedded microbial cells, making them resistant to antimicrobial agents [6]. An oral cavity serves as a perfect environment for biofilm formation due to its optimum temperature, pH, and nutrient supply. The accumulation and maturation of plaque over time can result in caries, gingivitis, and periodontitis [7].

Currently, the best method to maintain good oral health and prevent gingivitis and caries is a consistent oral hygiene habit that consists of brushing one’s teeth twice per day with a fluoride-containing toothpaste. Fluoride inhibits enamel demineralization and promotes remineralization by incorporating into apatite crystals, preventing caries formation [8]. Further improvements in plaque and gingivitis reduction can be achieved by brushing with oral hygiene products containing metal actives or quaternary ammonium compounds [9,10,11]. These compounds control the growth and accumulation of bacteria along various oral niches (i.e., teeth, gums, cheeks, and tongue) [12].

Stannous fluoride (SnF_2_) is one such antibacterial agent that has a long history of inclusion in toothpaste and mouthrinse formulations for anti-caries, erosion protection, hypersensitivity relief, and plaque and gingivitis control [13,14,15,16,17]. Formulations containing SnF_2_ have been around since the 1950s. The stability of the formulation has been significantly increased by the addition of 1% zinc phosphate [18]. Publications have reported the means by which SnF_2_ interrupts molecular processes, leading to gum inflammation and protecting enamel against acid erosion challenges [19,20]. There have been limited publications, however, on the effect of SnF_2_ on the composition, structure, and mechanical properties of the oral biofilm [21]. With the advent of advanced imaging systems and multi-omics approaches [22,23], researchers can better observe biological changes and gain new insights into the mode of action of the anti-bacterial agent. The application of atomic force microscopy (AFM) to study the biomechanical properties of multi-species biofilm have been reported previously [24,25,26]; however, few articles have provided information on the biomechanical properties of in situ-formed biofilm as a function of oral hygiene treatment. Similarly, confocal laser scanning microscopy provides a high-resolution view of biofilm architecture, thickness, and volume; moreover, when combined with Fluorescence In situ Hybridization (FISH), additional information can be acquired regarding the bacteria geographical distribution within plaque [27,28]. The FISH technique is limited, however, to the probes available. The 16S rRNA gene and metagenomic sequencing have similarly been developed to provide information on the composition of the microbiome down to species and, in some cases, strain level resolution [29,30]. Metatranscriptomic analysis further provides information regarding the gene expression of microbial species in a given environment. The objective of this study is to explore the impact of brushing with an SnF_2_-containing toothpaste on the biology and nanomechanical properties of oral biofilms. In order to achieve this goal, imaging and sequencing-based techniques have been used.

In this publication, we demonstrate the mode of action by which brushing with an SnF_2_-containing toothpaste controls the accumulation and growth of biofilm on oral surfaces. The results demonstrate why brushing with stable SnF_2_ formulations improves one’s oral health benefits beyond that which is obtained from traditional mechanical plaque removal via tooth brushing with a regular fluoride toothpaste.

## 2. Materials and Methods

A sequential monadic clinical study was conducted at the Colgate Technology Campus in Piscataway, NJ, USA. This study was approved by the US Institutional Review Board, Inc., Miami, FL, USA (IRB#-U.S.IRB2020CP/02). All participants provided written informed consent before participating in the study.

### 2.1. Inclusion Criteria 

Participants were included in the study if they met the following criteria: male or female, 18–65 years of age that were in good general health and available for the duration of the study; willing to sign the consent form and provide information on their medical history; willing to wear an intra-oral appliance, and had at least 20 natural teeth (excluding third molars and crowns).

### 2.2. Exclusion Criteria

Participants were excluded if they met the following criteria: were participating in another oral clinical study, exhibited an oral pathology, chronic disease, or had a history of allergy to oral or personal care products or their ingredients; used anticonvulsants, antihistamines, antidepressants, sedatives, tranquilizers, anti-inflammatory medication, or daily analgesics within one month prior to the start of the study or were scheduled to starting such intake during the course of the study; were pregnant or breastfeeding; used antibiotics or therapeutic mouthwash any time during or three months prior to entry into the study; ongoing use of medications known to affect the gingival tissues (i.e., calcium channel blockers, phenytoin, cyclosporine); evidence of periodontal disease (i.e., purulent exudate or tooth mobility); had periodontal treatment 12 months before the beginning of the study; were current smokers or had a history of alcohol or drug abuse; had 5 or more decayed untreated dental sites at screening; had a known salivary function impairment.

### 2.3. Study Visits and Clinical Procedures

Participants were first screened to assess whether they met the inclusion/exclusion criteria. At the screening, participants’ demographics and medical history were recorded, and an oral soft tissue examination was conducted. After signing an informed consent form, being provided a privacy notice, and meeting all inclusion requirements, participants were enrolled in the study. Thirteen participants were enrolled in the study. The participants were blinded to the products provided.

### 2.4. In Situ Model (Retainer with HAP Discs) Preparation and Design

**Figure 1:**
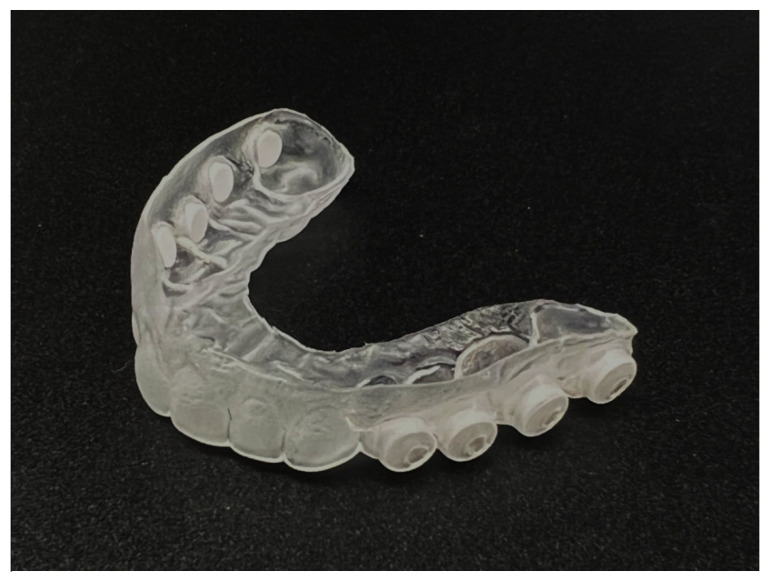
Photograph of the intra-oral retainer fitted with hydroxyapatite discs.

Maxillary impressions of each participant were taken by the dental hygienist. The impressions were poured with dental hard stone. The entire upper jaw’s retainer was fabricated with mouthguard material (Benco Dental, Pittston, PA, USA), of 0.040″X5″X5″clear thickness, using a heat/vacuum machine (Vacuum formers, Machine III, Keystone industries GmbH, Singen, Germany) and molded onto an impression where separate niches were created to fit hydroxyapatite (HAP) discs. Holes were made on the retainer at these sites to allow saliva flow and biofilm formation on the buccal side. Eight sterile hydroxyapatite (HAP) discs (5 mm in diameter, 2 mm thickness, HiMED Inc., Old Bethpage, NY, USA) were fitted (using sticky wax to secure placement) into the niches within the intra-oral retainer. Four HAP discs were seated at positions over the upper right premolars and molars and 4 over the upper left premolars and molars (Figure 1). A complete description of the device can be found in [29].

### 2.5. Intra-Oral Appliance Wear Instructions

Participants were instructed to wear the intra-oral appliance (Figure 1) for 48 h, only removing it during tooth brushing, eating, and drinking. Participants were told to insert the retainer immediately after tooth brushing and 30 min after eating and drinking.

### 2.6. Clinical Study Design

**Figure 2:**
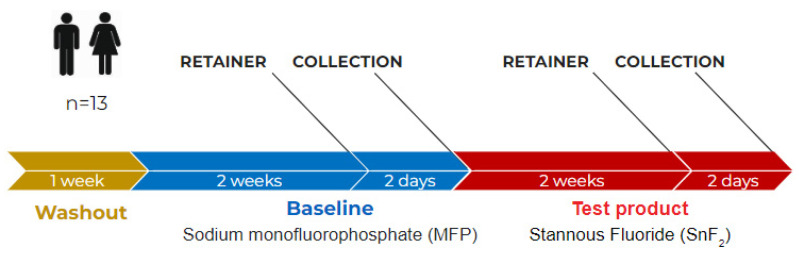
Schematic of the clinical study design.

The overview of the clinical study design is provided in Figure 2. After signing the informed consent form, participants were given a regular fluoride-containing toothpaste (Colgate Dental Cream (sodium monofluorophosphate (0.76%; 0.15% *w*/*v* fluoride ion), Colgate Palmolive Co, New York, NY, USA) and a commercial, soft-bristled manual toothbrush to use for 7 days prior to the start of the study. They were instructed to brush for two minutes, 2 times a day. They were asked not to use any oral rinse or oral hygiene product during this time period. Prior to the Baseline visit, they were told to refrain from any oral hygiene procedures for twelve hours prior to the examination and refrain from eating or drinking, except water, for four hours prior to the examination.

After 7 days, the participants returned to the clinical facility for their Baseline visit. During this visit, the soft and hard palatal mucosa, gingival mucosa, buccal mucosa, mucogingival fold areas, tongue, sublingual area, submandibular area, salivary glands, tonsil, and tonsillar and pharyngeal areas were examined and the results recorded.

Participants were then given another tube of the same regular F-containing toothpaste and were instructed to begin brushing for 2 min, 2 times a day with this product for the next 14 days. Prior to all subsequent visits to the clinic, participants were told to refrain from all oral hygiene procedures twelve hours prior to the examination and from eating or drinking (except water) for four hours prior to the examination. 

After the 2-week period, participants returned to the dental clinic and received an oral soft tissue exam. They were then given the intra-oral device fitted with 8 HAP discs. They were asked to continue to brush with the regular F-containing toothpaste. After 48 h, participants dropped off their retainers at the clinical site. They received a soft tissue assessment, and plaque was collected from the mandibular arch and stored at −80 °C for future microbiome analysis. Confocal and AFM measurements were initiated immediately for 2 of the 8 HAP discs present in the intra-oral device. One disc was used for confocal and another for AFM. The same disc position was used for the respective microscopy method across panelists for consistency. The remaining HAP discs were stored at −80 °C for future analysis. 

At this visit, the participants received an SnF_2_-containing toothpaste (Colgate Total^®^, Colgate Palmolive Co, New York, NY, USA) and were instructed to brush for 2 min 2 times a day for the next 14 days. The toothpaste contained 0.454% SnF_2_ (0.15% *w*/*v* fluoride ion). The participants were instructed not to use any other oral hygiene product during this time period. They returned to the clinic after 14 days, a soft tissue assessment was conducted, and they received their intra-oral device. The participants were given the same instructions, i.e., to wear the intraoral device for the next 48 h except while eating, drinking, and brushing their teeth. After the two day period, they returned their intra-oral device, and the clinical assessments, plaque collections, confocal microscope imaging, and AFM measurements were repeated. The study concluded at this visit.

### 2.7. Confocal Laser-Scanning Microscopy (LSM)

The in situ biofilms formed on the HAP discs were stained with 2.5 µM Syto 9 Green Fluorescent Nucleic Acid Stain (Invitrogen) for 15 min in dark as per manufacturer’s instructions. The discs were transferred to clean PBS in a glass-bottom plate, with biofilm facing towards the objective. Three-dimensional image stacks were collected from 5–6 fields of view selected randomly, with an inverted Nikon C2 inverted confocal microscope under 63X magnification. The data were quantified using Imaris 3D image processing software (Version 8.4, Bitplane, Oxford instruments, Zurich, Switzerland). Paired Student’s *t*-test was conducted using Minitab v.20, State College, PA USA, to compare the volume and thickness of the biofilms.

### 2.8. Atomic Force Microscopy (AFM) 

All topography measurements were performed in tapping mode at room temperature (25 °C) using a MultiMode 8 AFM with a NanoScope V controller (Bruker, Billerica, MA, USA). PeakForce QNM in contact mode was utilized for capturing high-speed data capture files. The AFM probe utilized in all imaging was the POINTPROBE-PLUS Silicon-SPM Sensor by Nanosensors. The characteristics of the implored force modulation probe include a resonant frequency of 45–115 kHz, a spring constant of 0.5–9.5 N/m, and a nominal radius of <10 nm. For each sample, three to five 25 µm × 25 µm regions on the HAP surface were randomly chosen for obtaining images, from which roughness values were extrapolated. High-speed data capture data were extracted at 5 µm × 5 µm scan sizes. Prior to imaging and analysis, the deflection sensitivity was calibrated on a sapphire substrate as well as on (polydimethylsiloxane) PDMS to validate the calibration. Although the spring constant of the probe was known, k was calibrated using the thermal tune method (Lorentzian air) and fit to achieve a value within 10% of the given value. Images were acquired with an aspect ratio of 1.00 and either 256 or 512 data points/line. Once images were obtained, processing was performed using the NanoScope Analysis Software v. 1.5 (Bruker, Billerica, MA, USA). All plane-fitting and image processing were consistent through all images analyzed (Plane-Fit and second order flatten) for obtaining roughness parameters, reporting the root mean square deviation from the mean (R_rms_) parameter for all data shown herein. For data extraction, force curves were taken per pixel and fit using Nanoscope Analysis Software v. 1.5, whereby the baseline for each force curve was corrected and the retract curve (which shows both peak force (maximum force) and adhesion force (minimum force) was fit using the Hertzian method Young’s modulus (GPa) and adhesion (nN) were obtained from pixel by pixel force curves, using Nanoscope v. 1.5 (Bruker Corporation). The Young’s modulus values were extracted using the Hertzian model for a spherical indenter, where force is related to the indentation depth from three equations [31]. The adhesion values were obtained from the minimum force and were also extracted through Nanoscope. The Hertz model is used as it has been extensively applied to biofilm mechanical analysis using AFM [25,26,32]. The raw data were assessed visually by making individual value box plots. Responses that exhibited skewness were given a log transformation to better approximate normality. Within subject product means, variances, and replicate sample sizes were computed. The replicate numbers varied for subjects. A weight factor for the ensuing ANOVA analysis was computed as the ratio of sample size to variance. Product differences were assessed by two-way weighted analysis of variance (ANOVA) with Subject and Treatment as main effect factors. A Treatment *p* value of 0.05 or less was used to indicate statistical significance. The statistical analysis was carried out using Minitab v.20.

### 2.9. DNA Extraction and 16S Amplicon Sequencing 

Genomic DNA was extracted from the biofilm samples using Qiagen DNeasy Power Biofilm Kit as per manufacturer’s instructions. DNA was eluted in a 25 μL elution buffer. PCR amplification of the 16S rRNA gene was carried out using the FastStart High Fidelity PCR system, Roche, Basel, Switzerland. The V3–V4 region was amplified using the 347F (5′-GGAGGCAGCAGTRRGGAAT-3′) and 803R primers (5′-CTACCRGGGTATCTAATCC-3′). The following PCR amplification conditions on the thermocycler were used: 1 cycle of 95 °C for 3 min; 25–30 cycles of 95 °C for 30 s, 60 °C for 45 s and 72 °C for 45 s; and 1 cycle at 72 °C for 5 min; final PCR product was held at 4 °C. Next, 2 μL of a mock community sample containing known bacterial composition was used as a positive control, and 2 μL of PCR-grade water was used as a negative control. The resulting PCR products were quality checked by gel electrophoresis (Agilent 7500 Kit, Agilent Technologies, Santa Clara, CA, USA) with expected DNA amplicons of approximately 540 bp. The sequencing libraries were prepared with dual indices using the Nextera XT kit (Illumina, San Diego, CA, USA) as per the manufacturer’s protocol. The DNA libraries were quantified using a PGloMax instrument (Promega, Madison, WI, USA) and Quant-iT dsDNA Assay High Sensitivity Kit (Invitrogen, Life Technologies, Carlsbad, CA, USA) following the manufacturer’s protocol. DNA samples were diluted down to a concentration of 4 nM with 10 mM Tris (pH 8.5). Prior to the sequencing run, DNA (5 uL) from each library, along with the 10% PhiX control, was pooled, mixed, and denatured. The 251 bp paired-end sequencing reactions were performed on Illumina MiSeq v3 Illumina, San Diego, CA, USA). Libraries were sequenced at the Hill’s Science and Technology Center (Topeka, KS, USA) using a MiSeq Reagent Kit v3 (Illumina, San Diego, CA, USA) (paired end, 250 cycles) on a MiSeq System (Illumina, San Diego, CA, USA). 

### 2.10. Bioinformatic Analysis for 16S rRNA Gene Amplicon Sequencing

The 16S rRNA gene sequencing data were analyzed with R (R Core Team 2021) in RStudio [33]. Primers were removed using CutAdapt v3.5 [34] and quality statistics were analyzed using FastQC v0.11.9 [35]. The data analysis was conducted using dada2 v1.20.0 pipeline [36]. Briefly, the sequencing reads were quality filtered and trimmed, dereplicated, denoised, merged, and filtered for chimeras; then, amplicon sequence variants (ASVs) were annotated against the expanded Human Oral Microbiome Database [37]. Decontam v1.12.0 [38] was used to remove contaminating reads identified in the negative water controls. Phyloseq v1.38.0 [39] functions were used for downstream analysis. Alpha diversity estimates, Shannon–Weaver and Simpson’s (1-D) Diversity Indices, and a number of observed ASVs were calculated with estimate_richness and vegan v2.5-7 [40]. The Shapiro–Wilk test was used to test for normalcy. Kruskal–Wallis Rank Sum Test statistic was calculated between treatments. Ordinations were constructed using PCoA with Bray–Curtis dissimilarity, Euclidean distance, and unweighted Unifrac distance measures to assess beta diversity. Analysis of variance was conducted using adonis2 in vegan on all three matrices. Alpha diversity, beta diversity, and taxonomy plots were built using phyloseq, ggplot2 v3.3.5 [41], color palettes from wesanderson v0.3.6 [42], and cowplot v1.1.1 [43]. Differential abundance was calculated using DESeq2 v1.32.0 [44] with poscounts estimation, local fit type, Wald significance test, and all other default options. Differential abundance was plotted with ggplot2 v3.3.5 [41].

### 2.11. Metatranscriptomics

Raw reads were quality checked using fastx-toolkit v0.0.14 [45]. Ribosomal RNA was removed using SortMeRNA v4.3.2 [46] against the ARB [45], Silva [47] databases for 16S bacteria, 16S archaea, 18S eukarya, 23S bacteria, 23S archaea and 28S eukarya, and RFAM 5S and 5.8S databases. Host reads were removed using Bowtie2 v2.4.2 [48], Samtools v1.11 [49], and BEDTools v2.30.0 [50] to align the reads to the human genome (GRCh38 RefSeq: GCF_000001405.39). Filtered reads were assembled using Trinity v2.1.1 [51], incorporating Trimmomatic v0.39 [52] and read normalization. Raw reads were aligned back to the assembled transcripts using Trinity’s built-in tool with bowtie2 and RNA-Seq by using the Expectation Maximization (RSEM) count estimates [53] estimation method. Transcripts were annotated using the Trinotate v3.2.1 [54] pipeline. Coding regions within transcripts were identified using TransDecoder v5.5.0 [55]. Transcript nucleotides and peptide sequences were annotated with blastx v2.11.0+ and blastp v2.11.0+ [56], respectively, against the UniProtKB Swiss-Prot database (downloaded on 21 April 2021; The UniProt Consortium, 2021) with an e-value cutoff of 0.001 Peptide sequences were searched for homology using HMMER v3.3.2 [57,58]. The location of transmembrane helices in the peptide sequences were predicted using TMHMM v2.0 [59]. Cross annotations were assigned using UniProtKB for KEGG [60], eggNOG [61], and Gene Ontology [62].

Transcript counts of the matrix and annotations were imported into R v4.1.0 [63] via RStudio v1.4.1106 [33] for analysis. Differential expression analysis was completed with NOISeq v2.36.0 [64] for treatments (noiseqbio function) and for individuals (noiseq function) with tmm normalization and gene length correction and default parameters for the remaining arguments and implemented functions from DESeq2 v1.32.0 [44] and edgeR v3.34.1 [65]. Statistical analyses were performed using R packages stats v4.1.0 [63], corrplot v0.92 [66], Hmisc v4.6-0 [67], FactoMineR v2.4 [68], and vegan 2.5-7 [40]. Interactive volcano plots were constructed with manhattanly v0.3.0 [69], and standard volcano plots were constructed with EnhancedVolcano v1.10.0 [70]. Additional plots were constructed using ggplot2 v3.3.5 [41] with functions from ggrepel v0.9.1 [71], ggpubr v0.4.0 [72], ggtern v3.3.5 [73], ggfortify v0.4.14 [74,75], and wesanderson v0.3.6 [42]. Housekeeping tools included the tidyverse v1.3.1 suite [76], tidyr v1.1.4 [77], reshape v0.8.8 [78], dplyr v1.0.7 [77], plyr v1.8.6 [79], and data.table v1.14.2 [80].

Gene set enrichment analysis was performed using GSEA v4.2.3 [81,82]. TMM normalized RSEM estimated gene expression aggregated by UniProt Entry ID and was profiled by treatment against a manually curated gene set database of Gene Ontology (GO) terms for each UniProt entry present in the dataset. Default parameters were used for GSEA without collapsing gene symbols. Parameters also included meandiv normalization mode, weighted enrichment statistics, and Signal2Noise ranking metric. Heatmaps were constructed using pheatmap v1.0.12 [83] for normalized expression data scaled by gene and ranked with GSEA. Detailed analytical methods can be found on GITHUB.

## 3. Results and Discussion

### 3.1. SnF_2_ Toothpaste Affects Biofilm Growth and Morphology

**Figure 3:**
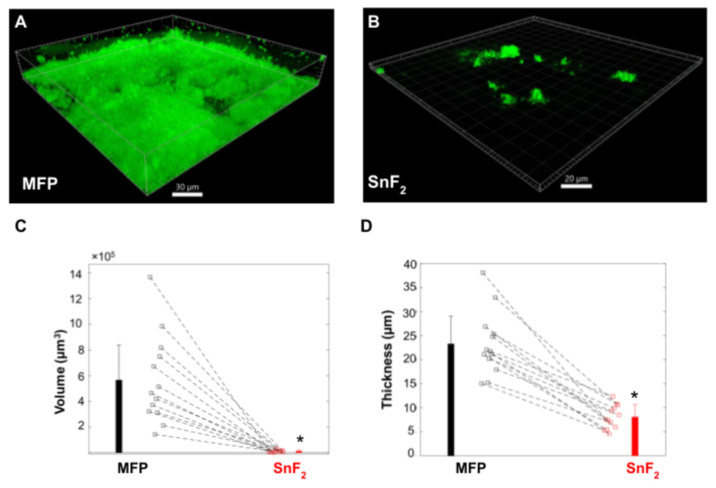
Confocal imaging and quantification of in situ biofilms: (**A**,**B**) 3D volume views of 2-day in situ biofilms formed with sodium mnofluorophosphate (MFP) and SnF_2_-containing toothpaste use for 14 days. (**C**,**D**) Plots of biofilm volume and thickness formed with MFP and Colgate Total SnF_2_ use. Three to four data points were collected for each panelist for each condition. The squares/points represent average value for each panelist per condition and bars represent average value of the data set per condition. The error bars represent standard deviation. The average volume and thickness values are significantly lower in the SnF_2_ group compared to that of MFP (* *p* < 0.00001, paired Student *t*-test).

All 13 participants successfully completed the study, and there were no adverse events reported. The differences in biofilm architecture after 14 days of brushing with the regular sodium fluoride-containing control toothpaste and the SnF_2_-containing test product are illustrated in Figure 3A,B. These example images are representative of what was observed for all of the study participants. There was consistently less biofilm observed after brushing with the SnF_2_ toothpaste. In addition, the biofilm was sparse, and bacterial microcolonies were clearly discernible. The volume and thickness of the biofilm were also calculated, and both measurements were significantly smaller for the biofilm formed after brushing with the SnF_2_-containing toothpaste (Figure 3C,D). The average values of biofilm volume decreased from 5.6 × 10^5^ µm^3^ to 1 × 10^4^ µm^3^ and that of thickness from 23.2 µm to 8.0 µm. These qualitative and quantitative findings illustrate that brushing with an SnF_2_-containing toothpaste stabilized with zinc phosphate, reduced biofilm growth, and modulated the architecture of the oral biofilm. The changes observed are due to the residual effects of the toothpastes retained on the oral surfaces, as the biofilms were not directly brushed with the toothpaste but were worn right after brushing the teeth with the toothpaste. This reflects the substantive nature of the toothpastes that protect teeth for prolonged hours. Alteration in the biofilm architecture is relevant for the penetration of antibacterial agents to reduce further biofilm growth. SnF_2_ has shown antibacterial properties previously in various types of in vitro and clinical studies [12,84,85]. The reduction in biofilm formation is likely due to decreased bioadhesion, and the anti-bacterial nature of the toothpaste A prior short duration (8 h) in situ study with SnF_2_ solution rinse has shown reduced adhesion and the initial colonization of oral bacteria on bovine enamel blocks [86]. Reduced adhesion to the dental surface and interference with initial colonization likely does not let the biofilm reach the maturation stage due to changes in gene expression profile. This impedes EPS formation, making biofilms less pathogenic [87]. The reduction in biofilm growth on HAP discs observed in the current study is due to the residual effect of the SnF_2_ toothpaste brushing of dental surfaces every 12 h and is suggestive of the effect on biofilm growth and morphology in a clinically relevant timeframe.

### 3.2. SnF_2_ Toothpaste Alters Nanostructure and Biomechanical Properties of the Biofilm

**Figure 4:**
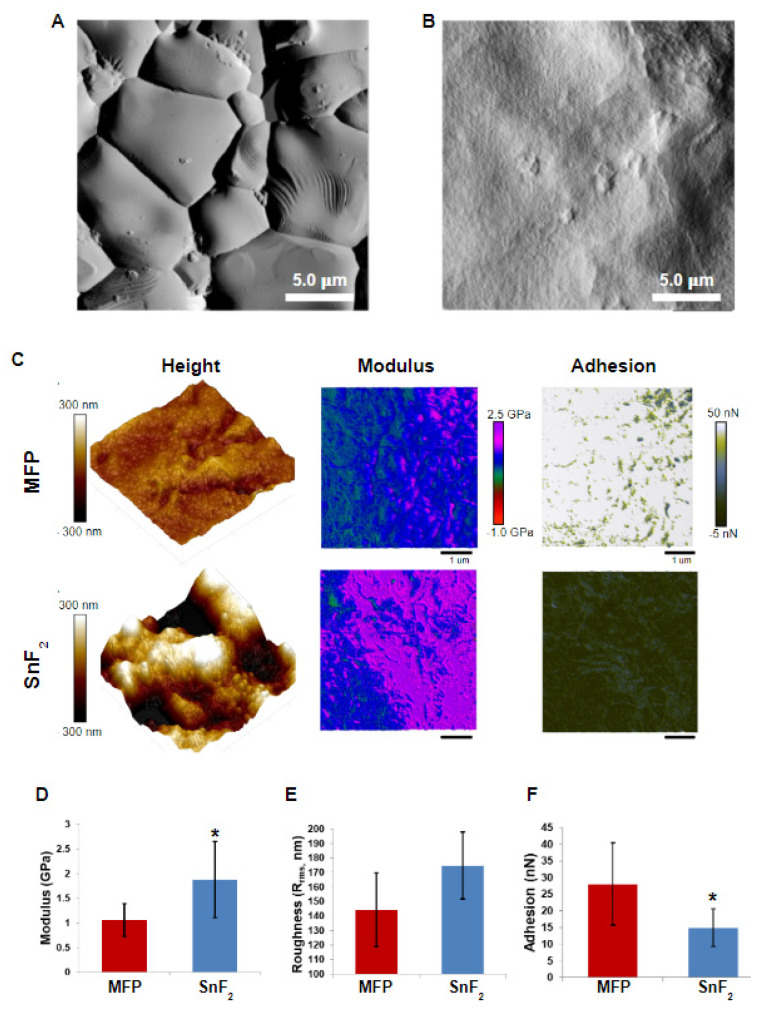
AFM images and quantification. PeakForce Error Images (25 μm × 25 μm) showing the surface of the hydroxyapatite (HAP) disc before (**A**) and after (**B**) biofilm growth. (**C**) HAP disc and biofilm height (left), qualitative modulus map (center) and qualitative adhesion map (right) for sodiummonofluorophosphate (MFP) (top) and SnF_2_ (bottom) biofilms. (**D**–**F**) Barplots showing quantitative comparison of Young’s modulus, root mean square height (roughness), and adhesion of MFP and SnF_2_ biofilms. All grouping information was calculated using Tukey method and 95% confidence interval, with asterisks indicating statistical significance with *p* < 0.05.

Using atomic force microscopy, or AFM, changes in surface roughness (R_rms_), Young’s modulus and adhesion (minimum force) properties of the biofilm were determined (Figure 4). The appearance of the HAP disc with and without biofilm formation is shown in Figure 4A,B. A significant increase in the overall Young’s modulus was observed for the biofilm generated after brushing with the SnF_2_ toothpaste compared to that of the fluoride toothpaste (Figure 4C,D). There was a directional increase in roughness for the SnF_2_ biofilm (Figure 4E), seemingly caused by a shift in biofilm architecture and growth tendencies from an overall more uniform biofilm coating to that dominated by the presence of discrete bacterial microcolonies. The maximum adhesion point (pull-off, minimum force) after brushing with the SnF_2_ dentifrice was significantly lower (Figure 4C,F), an indication that brushing with this product has an effect on the local nanomechanical properties. The observed changes in the biomechanical properties of the biofilm are generally attributed to the overall morphology and distribution of biofilm, as well as to changes in the biochemical composition of the biofilm matrix and cell wall [88,89]. Changes in the composition and distribution of the EPS matrix have been previously associated with reduced adhesion with the application of other oral care products [24]. EPS formation is a complex process that is controlled by a large number of genes [90,91]. The observation in changes in biophysical properties is likely an outcome of the effect of SnF_2_ on bacterial gene expression associated with biofilm formation. To the authors’ knowledge, this is the first report demonstrating anti-adhesion properties of SnF_2_-containing toothpaste. The decrease in adhesion properties is highly relevant for oral biofilms. Less adhesive and stiffer biofilms imply that they can more easily be removed by brushing compared to the more adhesive biofilms. Additionally, less adhesive biofilms have been shown to be less virulent because of their inability to form robust biofilms [88].

### 3.3. SnF_2_ Toothpaste Maintains the Natural Healthy Oral Microflora

**Figure 5:**
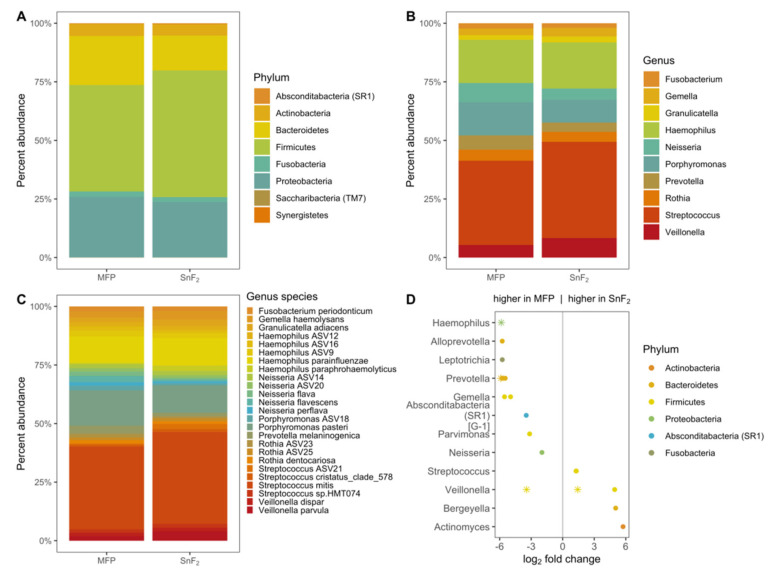
Oral bacterial community composition with the use of sodium monofluorophosphaste (MFP) and SnF_2_-containing toothpastes. Stacked bar plots showing relative abundance of taxa at phylum (**A**), genus (top 10) (**B**), and species (top 25) (**C**) levels in biofilms formed with MFP and SnF_2_-containing toothpaste use. (**D**) Plot showing differential abundance of taxa at the genus level. The genera represented higher in MFP and SnF_2_ biofilms, which are shown on the left and right side of the central line, respectively. Only genera with *p* < 0.1 are plotted and significantly different genera (*p* < 0.05) are denoted as asterisks (Wald significance Test).

To examine the effects of fluoride and SnF_2_-containing toothpastes on oral microbial taxonomic diversity, three HAP discs with in situ formed biofilms were pooled for each of the 13 individuals for 16S rRNA gene amplicon sequencing. A summary of the amplicon data can be found in Table 1 and counts of amplicon sequence variants (ASVs) identified in each sample, along with taxonomic annotation, are provided in Supplementary Materials Table S1. A total of eight phyla were identified in 26 samples. The top phyla observed in each category are Fir-micutes, Proteobacteria, Bacteroidetes, and Actinobacteria (Figure 5A). Relative abundances of top genera detected are *Streptococcus* (MFP 34.4%, SnF_2_ 39.2%), *Haemophilus* (MFP 17.6%, SnF_2_ 18.8%), *Porphyromonas* (MFP13.5%, SnF_2_ 9.3%), *Neisseria* (MFP 7.8%, SnF_2_ 4.5%), and *Prevotella* (MFP 5.8%, SnF_2_ 3.7%) (Figure 5B). Of the 85 total genera observed in this study, only a few contained species that were significantly different in their abundance between the two treatment groups. There is a ~64-fold reduction in the abundance of species within the disease-associated genera, *Haemophilus* sp. and *Prevotella* sp. (Figure 5D, Table 2, Supplementary Materials Table S2) in the SnF_2_ group compared to that of the control toothpaste, and a 10.5-fold reduction in *Veillonella* sp.

**Figure 6:**
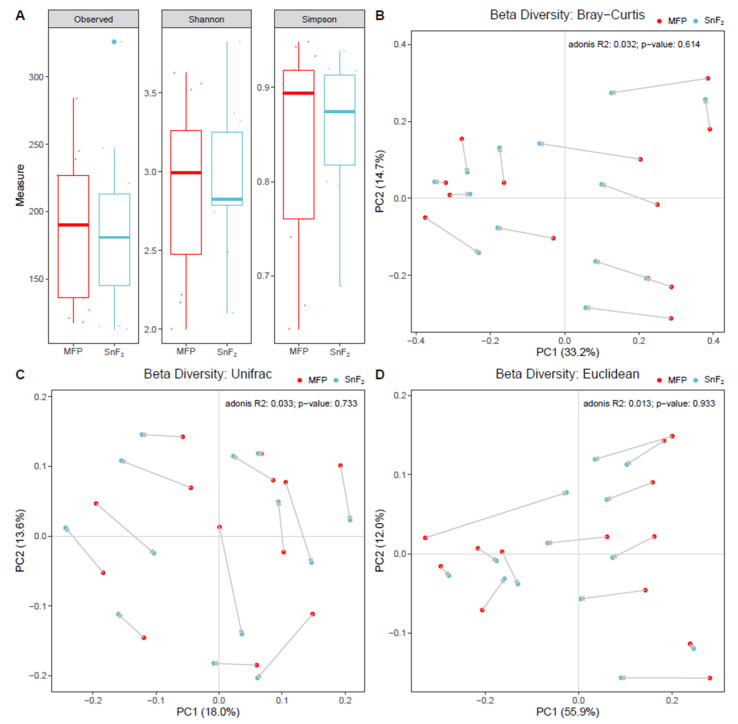
Diversity measurements of oral bacterial community with the use of sodium monofluorophosphate (MFP) and SnF_2_-containing toothpastes: (**A**) Boxplots showing alpha diversity of bacterial communities by observed ASVs and measured Shannon and Simpson indices. Each point in the plot is one sample, top and bottom of the box represent upper and lower quartiles, the line is at median, and bars show 95% confidence interval. (**B**–**D**) Beta diversity Principal Component Analysis (PCoA) plots showing distances of microbial communities in MFP and SnF_2_ samples measured using Bray–Curtis, Euclidean, and unweighted-unifrac distance matrices. Each dot represents a sample. R2 and *p* values calculated by Adonis test for permutational multivariate analysis of variance using distance matrices are shown on the top right of each beta diversity plot.

The diversity of the organisms detected within a sample was measured for richness and evenness using Shannon and Simpson’s diversity indices. The mean number of recovered amplicon sequence variants (ASVs) decreased after the use of SnF_2_, though the distribution was highly variable (Figure 6A). The differences between group diversity matrices are non-significant (Kruskal–Wallis rank sum test, *p* = 0.7004 for observed, *p* = 0.9387 for Shannon, and *p* = 0.9387 for Simpson). The comparison of diversity across treatment groups was carried out using Bray–Curtis, Euclidean, and unweighted-Unifrac beta diversity matrices. The regular fluoride and SnF_2_ samples are fairly well-distributed in space and do not form any separate clusters (Figure 6B–D). Combining observations from alpha and beta diversity matrices, the use of SnF_2_ toothpaste did not cause significant changes in the diversity of the natural microbiome. There were some changes in relative abundances of certain taxa; however, none were completely eliminated. This is mainly because only healthy individuals with healthy oral microflora were included in this study. Changes in microbial community composition are, however, expected in diseased populations, as SnF_2_ is known to affect the growth of disease-associated bacteria [92]. The ability of SnF_2_ to maintain the natural balance of oral bacteria in healthy individuals indicates that it has broad-range antibacterial properties that impact all bacteria and control the overall growth of biofilm in healthy individuals. Similar results were obtained for the salivary microbiome after the long-term use of SnF_2_ in two separate clinical studies [93,94]. Hence, the healthy natural microbiome remains stable with the use of SnF_2_ products.

### 3.4. Gene Expression of Supragingival Plaque

The effect of SnF_2_ on bacterial gene expression was detected with metatranscriptomics. RNA was sequenced from supragingival plaque samples of six panelists after MFP and SnF_2_ use for 14 days. A total of 398,245,418 paired-end reads (796 million total) were sequenced with a mean read length of 117 bp. The mean number of sequences per sample was 33,187,118 pairs, ranging from 22,893,645 to 47,615,860 reads. After rRNA removal (mean 48.04% of reads removed), host read removal (mean 2.17% removed), and quality trimming (mean 23.02% removed), the average reads remaining per sample for downstream analysis was 11,684,318 or 34.04%. The remaining reads were co-assembled into 2,685,947 transcripts (minimum length 150 bp), and 652,963 were removed with no reads aligned to them. Of the raw reads, 176,850,832 were aligned to transcripts, 151,816,649 were assigned 67,482 UniProt Entry IDs, and 105,811,801 were assigned 7734 KEGG Orthologies (KO).

**Figure 7:**
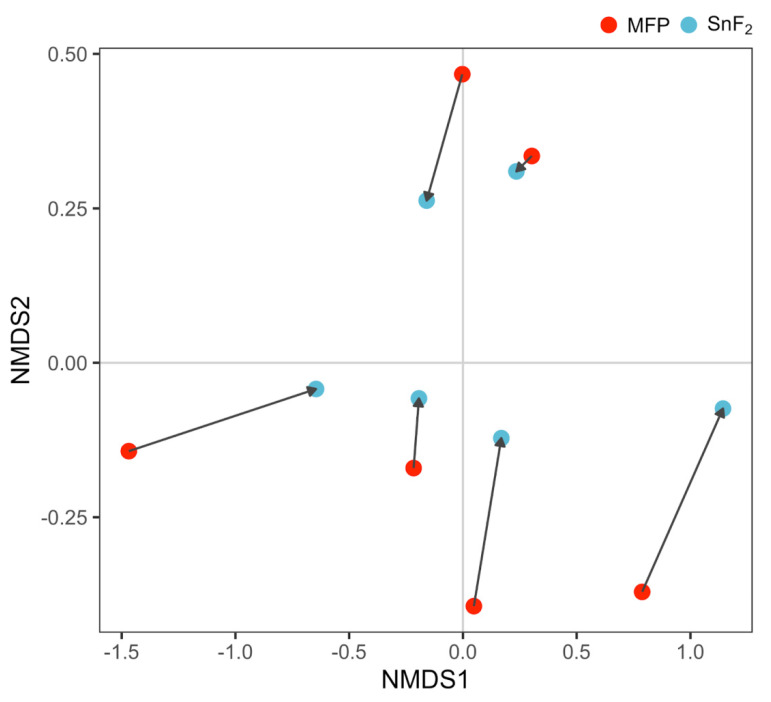
Non-metric multidimensional scaling (NMDS) plot showing distribution of panelists based on Bray–Curtis dissimilarity matrix calculated from TMM normalized transcript count estimates. Sodium monofluorophosphate (MFP) and SnF_2_ samples from the same panelist are connected by an arrow.

Samples were compared using non-metric multidimensional scaling (NMDS) on normalized RSEM count estimates (Figure 7). Functional genes were aggregated by KO for each sample and compared with NMDS (Supplementary Materials Figure S1). At the transcript level, variation decreased in the SnF_2_ group, with coordinates becoming closer together; however, variation remained high enough that the MFP and SnF_2_ groups remained statistically indistinguishable (F-statistic: 0.213, *p*-value: 0.654). Examining the data at the KO level shows reduced overall variation; however, generally, the same trend of higher variation in the MFP group makes comparison between groups statistically insignificant (F-statistic: 0.201, *p*-value: 0.664). There are changes in gene expression at the individual level (shown by arrows in Figure 7 and Supplementary Materials Figure S2); however, similar to the bacterial community structure (Figure 6C,D), gene expression profiles for an individual are more similar to themselves before and after treatment than to a treatment group. High variability in amplicon and gene expression data can be attributed to a large number of factors, such as diet, smoking habit, lifestyle, health, and genetics, and can be challenging to control in clinical studies. Smaller sample size and a lack of biological replicates are other reasons for higher variability that can be dealt to some extent by recruiting more participants and collecting multiple samples from the same participant in the future studies.

**Figure 8:**
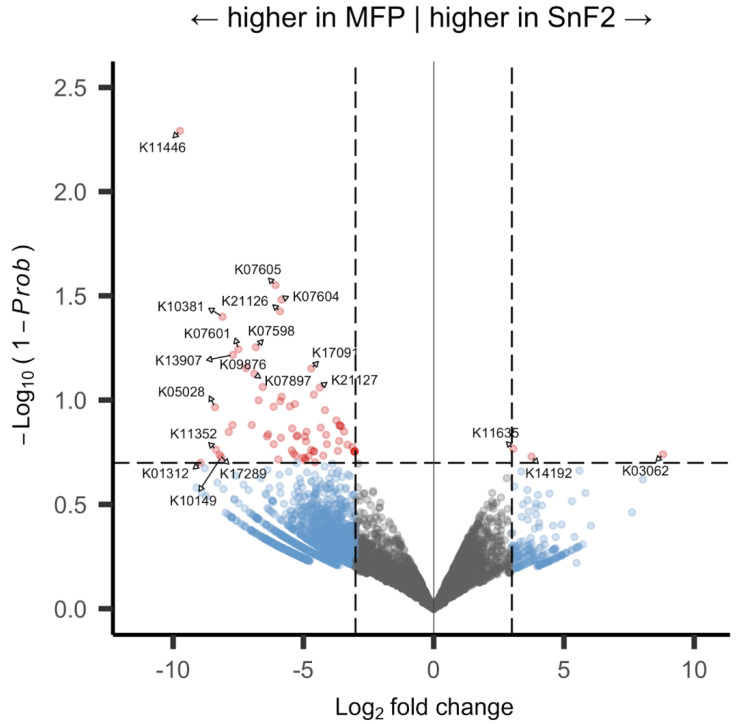
Volcano plot for differential expression of SnF_2_ vs. sodium monofluorophosphate (MFP) treatment: points are labeled with KEGG Orthology (*n* = 7734), grey pointd are not significant, blue have a log2 fold change greater than 3, yellow have a 1-prob < 0.2, and red have both a high fold change and significant probability; fold change and probability cutoffs are marked with dotted lines.

Differential gene expression of KOs between control and SnF_2_ groups and for individual panelists is visualized in volcano plots (Figure 8 and Supplementary Materials Figure S2, respectively). Of the 7734 KOs, 71 were significantly differentially expressed with a probability cutoff of 0.8 without biological replicates; 68 were higher in control, and 3 were higher in the SnF_2_ group (Figure 8). Further analysis of the gene expression revealed pathway and gene level patterns which reflected the reaction of the microbiome to SnF_2_.

**Figure 9:**
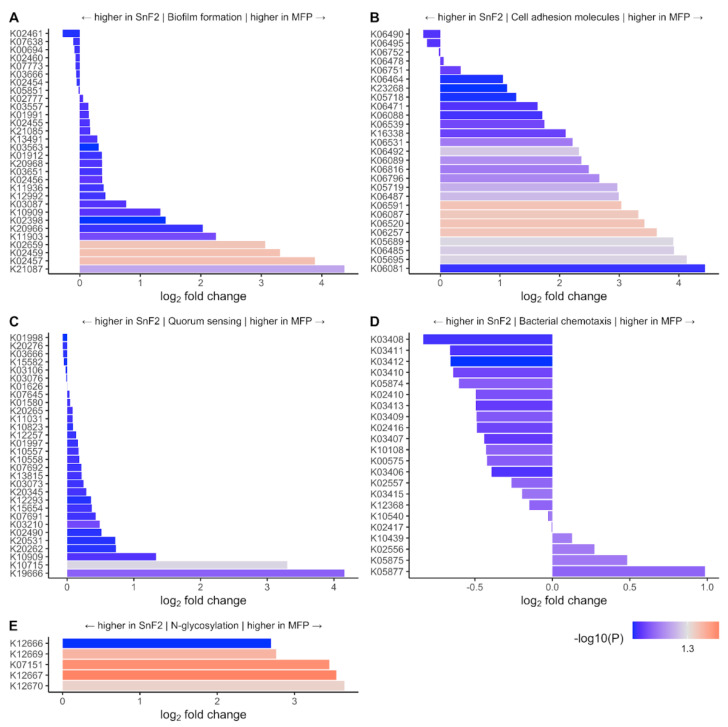
KEGG pathway analysis for the identification of differentially expressed KEGG orthologs. Plots are up to the 30 most significant KOs in each pathway (**A**) Biofilm formation including KEGG pathways map02025, map02026, and map05111; (**B**) Cell adhesion molecules pathway map04514; (**C**) Quorum sensing pathway map02024; (**D**) Bacterial chemotaxis pathway map02030; and (**E**) N-glycosylation by oligosaccharyltransferase module M00072.

In order to identify pathways impacted by the use of SnF_2_ toothpaste, the differentially expressed gene sets were mapped to KEGG pathways. Interestingly, pathways associated with biofilm formation, cell adhesion, quorum sensing, and N-glycosylation had significantly lower expression in the SnF_2_ group compared to the control group (Figure 9 and Supplementary Materials Table S3). Pathway level analysis of the gene expression is consistent with the microscopy observations (Figure 3 and Figure 4). Confocal microscopy findings revealed greatly reduced biofilm formation for the treatment group, and AFM microscopy findings illustrated a reduction in biofilm adhesion. 

**Figure 10:**
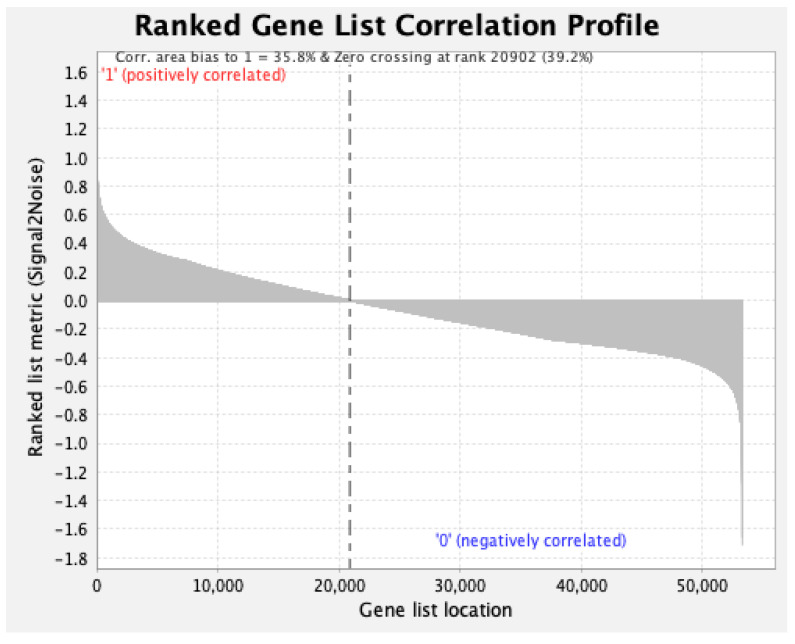
Plot showing correlation between ranked gene list and toothpaste treatment where ‘1’ represents SnF_2_ and ‘0’ represents sodium monofluorophosphate (MFP). Out of the 53,290 total genes, 20,902 correlated with SnF_2_ while 32,388 correlated with MFP.

**Figure 11:**
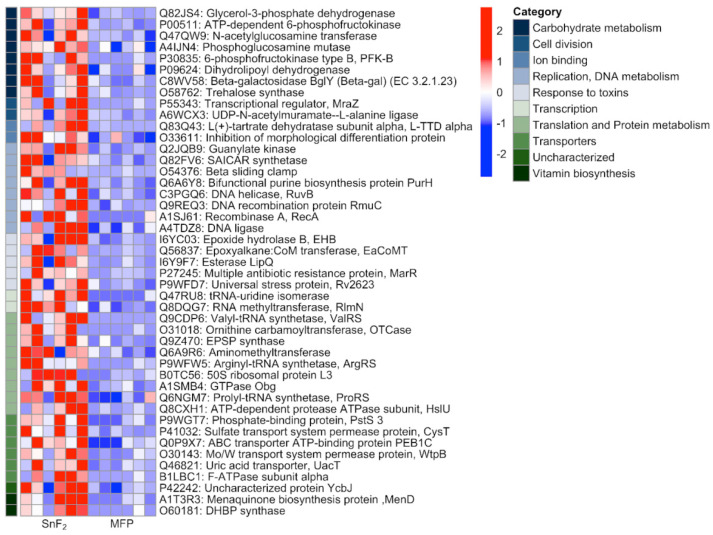
Heat map showing gene expression values of top genes correlated with SnF_2_. Range of colors (red, pink, light blue, dark blue) shows the range of expression values (high, moderate, low, lowest).

Correlations between individual genes and the two toothpastes used in the study were further explored using GSEA [81]. The entire dataset comprised 53,290 genes. Out of these, 20,902 (39.2%) correlated with SnF_2_, while 32,388 (60.8%) correlated with the control (Figure 10). The full list of genes with Uniprot IDs along with their rank metric score is provided in Supplementary Materials Table S4. Expression levels of top genes correlated with SnF_2_ are shown in Figure 11. A large number of genes are involved in basic cellular processes, such as, carbohydrate, protein, and nucleotide metabolism (Figure 11). Interestingly, cell wall-associated proteins were found to be highly correlated with the SnF_2_ group. This includes MraZ, a highly conserved DNA-binding transcription regulator [95]. It is a negative regulator of the bacterial cell division and cell wall (dcw) cluster [96]. In this cluster, peptidoglycan biosynthesis genes are located towards the 5′ end, and important cell division factors such as ftsz are located towards the 3′ end. Studies in *E. coli* and *Bacillus subtilis* have shown that overexpression of MraZ is lethal due to the inhibition of cell division [95,96]. This points towards a possible novel function of SnF_2_ for biofilm control by the inhibition of cell wall formation and cell division.

Another group of genes identified to be highly correlated with the SnF_2_ group were part of the ATP-binding cassette (ABC) transporter system [97]. These are multi-protein complexes present in the cell membranes for the import and export of molecules from the cell. These transporters import hydrophilic molecules and export lipids, steroids, metabolites, and toxins [97]. ABC transporters are a large group of proteins with multiple functions. In *Listeria monocytogenes*, these are negative regulators of biofilm formation, likely working through the release of anti-biofilm formation signaling molecules [98]. Intriguingly, other functions of ABC transporters include their role in cell wall biosynthesis. In *Bacillus subtilis*, overexpression of the YtrBCDEF ABC transporter leads to the production of a thicker peptidoglycan layer. This has negative physiological implications, such as the loss of genetic competence for the uptake of foreign DNA and biofilm formation defects [99]. The biofilms formed have altered morphology, likely due to increased thickness of the cell wall. This could be the reason for the changes in nanomechanical properties observed by AFM in the current study. AFM is an excellent tool for studying cell surface topographies and has been extensively used for studying changes in cell wall architecture in response to antimicrobials [100]. Further exploration of the cell wall at the biochemical and molecular levels will expand our understanding of biomechanical changes observed in response to SnF_2_.

**Figure 12:**
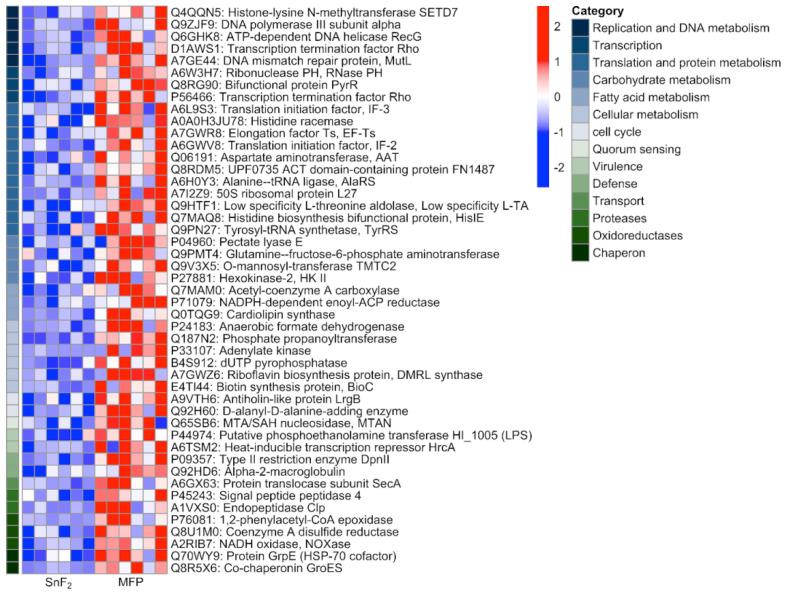
Heat map showing gene expression values of top genes correlated with sodium monofluorophosphate (MFP). Range of colors (red, pink, light blue, dark blue) shows the range of expression values (high, moderate, low, lowest).

Next, genes correlated with the control toothpaste group were explored. These are the genes which are over-expressed in control biofilms and under-expressed in the SnF_2_ group. Expression levels of top genes correlated with the control biofilm are shown in Figure 12. These include several genes associated with biofilm formation and maintenance. Proteins associated with DNA replication, transcription, and translation were upregulated, suggesting bacterial growth. A large number of carbohydrate metabolism proteins were observed that support cell wall formation and bacterial growth. One such protein is Pectate Lyase (EC 4.2.2.), a glycan metabolism protein [101]. It is known to have a commensal effect of degrading pectin from ingested plant-based food to provide a source of carbon for bacterial growth [102]. It is a well-studied enzyme in gut microflora and is of special importance to vegetarians who depend on their microflora for the digestion of pectin [102]. The identification and upregulation of this protein in oral biofilm suggests a similar function in the oral cavity for digesting food bound to the dental surface to support bacterial growth in dental plaque.

Another protein associated with biofilm establishment and maintenance identified to be under-expressed in SnF_2_ was LrgB, an Antiholin-like protein [103]. It is a programmed cell death pathway protein. In *Staphylococcus aureus*, *lrg* and *cid* operons work in an antagonistic manner to regulate cell death and lysis [103]. These two operons play an important role in biofilm formation by controlled cell lysis for the release of genomic DNA. The released DNA is integrated into the biofilm matrix as extracellular DNA and provides structural stability to the biofilm matrix. The eDNA is essential for the establishment, maintenance, and perpetuation of biofilms. It also protects the biofilm from cationic antimicrobials by chelation because of its anionic nature [104]. Reduced expression of the *LrgB* gene in the SnF_2_ group provides reasoning for diminished biofilm establishment at the early stages and reduced structural stability in the established biofilms. 

In addition to the availability of resources, communication between bacteria is also crucial for successful biofilm formation. In this study, the expression pattern of 5′-methylthioadenosine/S-adenosylhomocysteine nucleosidase (MTAN) was high and positively correlated with the control group. MTANs are bacterial enzymes that are part of the S-adenosylmethionine-related quorum sensing pathways. MTANs are directly involved in the biosynthesis of two classes of auto-inducers, AI-1 and AI-2 [105]. These signaling molecules are produced by the bacteria for communication with each other and for coordinating gene expression for a given environment. This induces biofilm formation and increases virulence factor production. Quenching of these quorum-sensing molecules disrupts microbial communication and prevents biofilm formation [106]. Much higher expression of quorum sensing-genes in the control group explains robust biofilm, while reduced quorum sensing in the SnF_2_ group explains the obstruction in the biofilm formation, as observed through confocal microscopy (Figure 3).

In addition to biofilm establishment and quorum sensing, biofilm protection is also critical for biofilm maintenance. Such genes were found to be under-expressed in SnF_2_ compared to control biofilms. Alpha-2-macroglobulin proteins are known to counteract the anti-biofilm properties of host proteases [107]. They protect the bacterial cell by trapping a broad range of proteases through a covalent interaction with an activated thioester. This acts as the innate immune response of eukaryotes for protecting cellular integrity [107]. Another interesting protein overexpressed in the control biofilms is HrcA, a heat inducible transcription repressor which negatively regulates class I heat shock operons (grpE-dnaK-dnaJ and groELS) [108]. HrcA is also a regulon which regulates the expression of several other cellular functions unrelated to heat shock response, such as bacterial adhesion, motility, survival, and virulence [109]. Studies in *Helicobacter pylori*, a gastric human pathogen, have revealed that *HrcA* is upregulated in biofilms and influences biofilm formation and motility [110]. Similar observations have been regarding *Listeria monocytogenes*, wherein HrcA, along with DnaK are shown to be important for biofilm formation as well as for protection against benzalkonium chloride and peracetic acid antibiofilm agents [111]. The reduced expression of these genes in the SnF_2_ group is indicative of biofilms being more vulnerable to host defense mechanisms and the altered biofilm properties of adhesion and motility.

Besides biofilm-associated genes, toxins and toxin-secretion proteins were also negatively correlated with SnF_2_ biofilms. An interesting finding is the reduced expression of the lipopolysaccharide (LPS) core region biosynthetic process gene, Phosphoethanolamine transferase. LPS is a component of the outer membrane of gram-negative bacteria and is a well-known endotoxin responsible for a large number of inflammatory diseases [112]. This protein and the associated pathway are not enriched in SnF_2_ samples, suggesting that SnF_2_ reduces the production of endotoxins in biofilms. This is crucial for host soft tissue health and supports previous findings from in vitro and clinical studies [113,114].

A related protein, SecA, of the Sec secretion pathway, was also found to be negatively correlated with SnF_2_. The Sec secretion pathway is a highly conserved mechanism of protein export found in all classes of bacteria and comprises multiple Sec proteins [115]. SecA is an ATPase in the protein translocation machinery that plays a vital role in the secretion of proteins, toxins, and other virulence factors. It is essential for bacterial survival and enhances the attachment of bacteria to eukaryotic cells. In addition, it promotes the virulence of bacterial pathogens [115]. Negative correlation of this pathway with SnF_2_ suggests that use of SnF_2_ reduces the production as well as release of virulence factors.

In summary, metatranscriptomics has provided a comprehensive view of the effect of SnF_2_ on gene expression in oral biofilms. A large number of genes associated with biofilm establishment, maintenance, propagation, and virulence were downregulated with the use of SnF_2_. This is a novel finding for this product and provides insights into the mode of action of SnF_2_ for biofilm control.

## 4. Conclusions

This clinical study has explored the anti-biofilm benefits of brushing with an SnF_2_-containing toothpaste. We have provided microscopy, biomechanical, and gene expression-based findings illustrating the mechanisms by which SnF_2_ controls biofilm accumulation. The multiple approaches used in this study complement each other and strengthen the evidence the anti-biofilm properties of SnF_2_. Control of biofilm growth is an important first step towards maintaining eubiosis and a healthy mouth. SnF_2_-containing toothpaste changes the biofilm architecture and gene expression, making the biofilm less adhesive and non-virulent. This creates a less pathogenic environment for teeth and gums. Since poor oral health can lead to systemic bacteria exposure and inflammation, maintaining a healthy oral microbiome is critical to minimize poor overall health outcomes [116,117,118]. The findings reported in this manuscript describe how stabilized SnF_2_ formulations are effective in helping people maintain a healthy oral flora.

## Figures and Tables

**Table 1:** Summary statistics of the 16S rRNA gene amplicon data.

Group	Sodium Monofluorophosphate	SnF_2_
No. of reads (mean)	199,766	214,510
No. filtered reads (mean)	146,682	155,574
Total ASVs	1034	1026
Unique ASVs	450	442
ASVs ≥ 10	692	712
Mean ASVs	186	185
±sd	±54.6	±59.9
Shannon diversity index	2.91	2.97
±sd	±0.548	±0.437
Simpson’s diversity index	0.842	0.861
±sd	±0.107	±0.069

**Table 2:** List of differentially abundant taxa observed in SnF_2_ vs. sodium monofluorophosphate (MFP) populations.

Taxa ID	Phylum	Genus Species	log2 FC	*p*-Value
ASV11	Firmicutes	*Veillonella parvula*	1.427	0.039 *
ASV13	Proteobacteria	*Neisseria flavescens*	−1.976	0.054
ASV45	Absconditabacteria (SR1)	*Absconditabacteria* (SR1) [G-1]	−3.471	0.060
ASV50	Firmicutes	*Streptococcus sanguinis*	1.267	0.097
ASV73	Firmicutes	*Streptococcus* sp.	1.296	0.093
ASV98	Firmicutes	*Veillonella* sp.	−3.435	0.048 *
ASV106	Proteobacteria	*Haemophilus* sp.	−5.845	0.047 *
ASV118	Bacteroidetes	*Prevotella* sp.	−5.882	0.046 *
ASV122	Firmicutes	*Gemella* sp.	−5.538	0.060
ASV125	Bacteroidetes	*Alloprevotella* sp. HMT914	−5.755	0.051
ASV126	Bacteroidetes	*Prevotella aurantiaca*	−5.472	0.063
ASV128	Firmicutes	*Parvimonas* sp.	−3.147	0.073
ASV185	Firmicutes	*Gemella* sp.	−4.961	0.092
ASV190	Firmicutes	*Veillonella* sp.	4.944	0.093
ASV204	Bacteroidetes	*Bergeyella* sp.	5.039	0.087
ASV235	Bacteroidetes	*Prevotella* sp.	−5.760	0.050
ASV242	Actinobacteria	*Actinomyces* sp.	5.742	0.051
ASV325	Fusobacteria	*Leptotrichia* sp.	−5.749	0.051

* Statistically significant with *p* < 0.05.

## Data Availability

The raw sequence data presented in this study are available on NCBI with BioProject id PRJNA855131. The code for metatranscriptomic analysis is available at https://github.com/alouyakis/snf2.

## Supplementary Materials

The following supporting information can be downloaded at: https://www.mdpi.com/article/10.3390/microorganisms10091691/s1, Figure S1: Non-metric multidimensional scaling (NMDS) plot showing distribution of panelists based on Bray–Curtis dissimilarity matrix calculated from TMM normalized aggregated KEGG Orthologies. Sodium monofluorophosphate (MFP) and SnF_2_ samples from the same panelist are connected by an arrow; Figure S2: Volcano plots for differential expression (SnF_2_ vs. MFP) of each panelist before and after treatment for samples (A) 1 vs. 14, (B) 4 vs. 17, (C) 8 vs. 21, (D) 10 vs. 23, (E) 11 vs. 24, and (F) 12 vs. 25; points are labeled with KEGG Orthology, grey are not significant, blue have a log_2_ fold change greater than 3, yellow have a 1-prob < 0.05, and red have both a high fold change and significant probability; fold change and probability cutoffs are marked with dotted lines. Note—probability is not based on replication. Table S1: Identified ASVs with counts for each sample and taxonomic annotations. Table S2: Differential abundance of ASVs in SnF_2_ vs. Sodium monofluorophosphate (MFP) populations. Table S3: Differential expression of KEGG pathways. Table S4: Rank ordered list of all genes.
