# Supplementary material for: Effect of a Stannous Fluoride Dentifrice on Biofilm Composition, Gene Expression and Biomechanical Properties
Source: Microorganisms. 2022 Aug 23;10(9):1691. doi: 10.3390/microorganisms10091691 (PMC9506307; doi:10.3390/microorganisms10091691)
Supplement: Supplementary file 1 [file microorganisms-10-01691-s001.zip › Supplementary Figures.pdf]

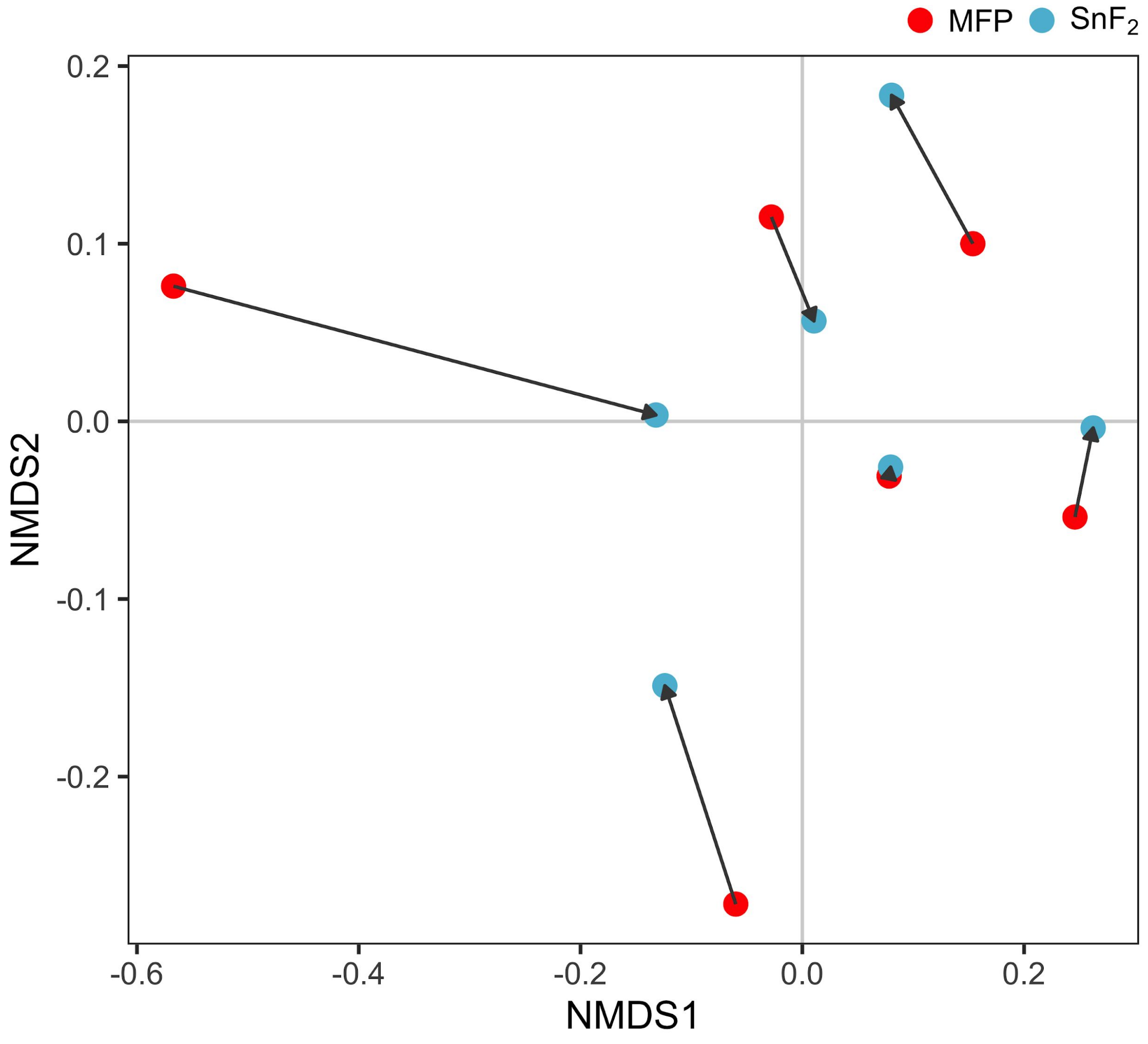

Figure S1. Non-metric Multidimensional Scaling (NMDS) plot showing distribution of panelists based on Bray-Curtis dissimilarity matrix calculated from TMM normalized aggregated KEGG Orthologies. MFP and SnF_2 samples from the same panelist are connected by an arrow.

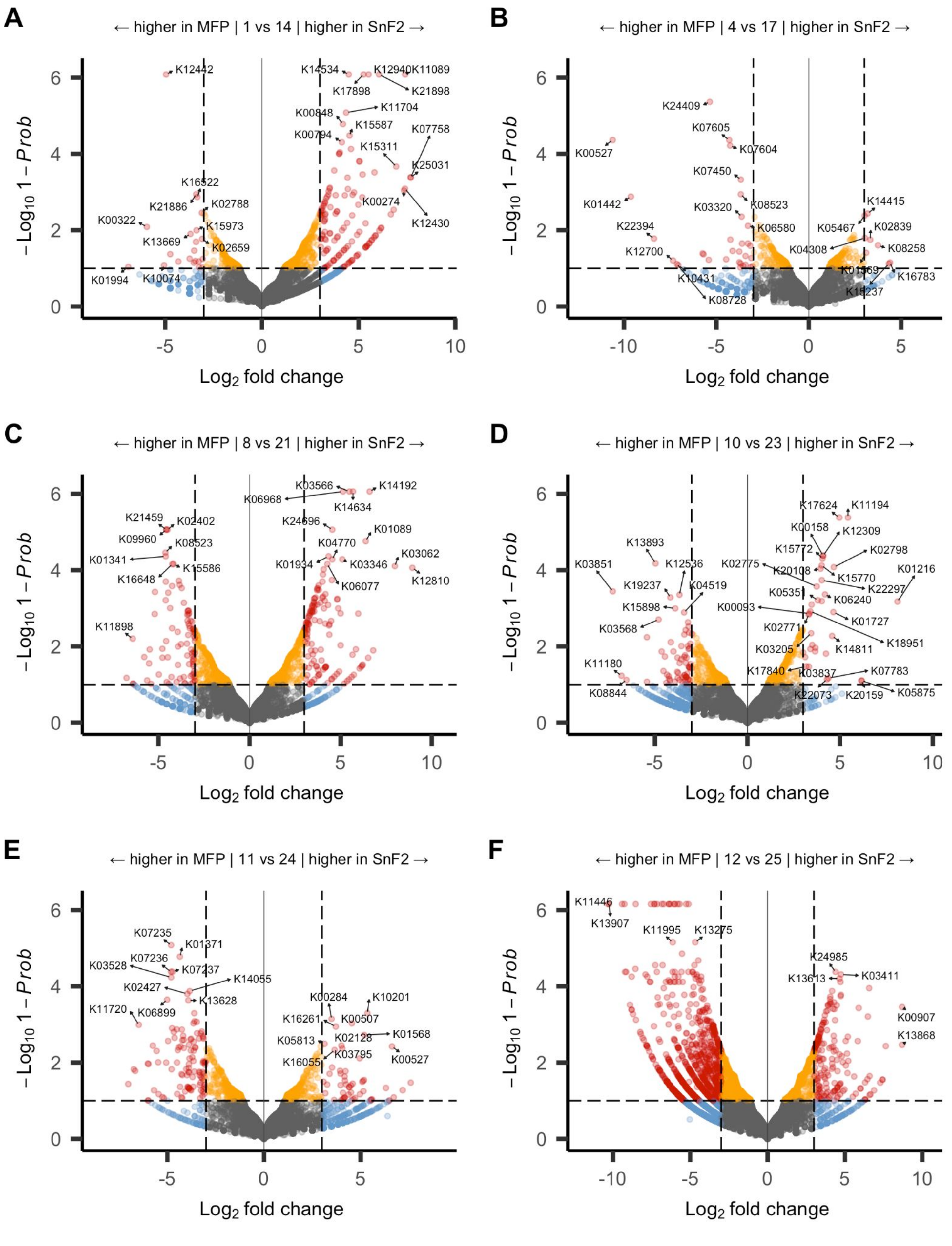

Figure S2. Volcano plots for differential expression (SnF_2 vs. MFP) of each panelist before and after treatment for samples A) 1 vs 14, B) 4 vs 17, C) 8 vs 21, D) 10 vs 23, E) 11 vs 24, and F) 12 vs 25; points are labeled with KEGG Orthology, grey are not significant, blue have a $\log_2$ fold change greater than 3, yellow have a 1-prob < 0.05, and red have both a high fold change and significant probability; fold change and probability cutoffs are marked with dotted lines. Note - probability is not based on replication.
